# Mapping the certolizumab pegol epitope on TNF and comparison with infliximab, adalimumab and etanercept

**DOI:** 10.1186/1479-5876-9-S2-P43

**Published:** 2011-11-23

**Authors:** Alistair I Henry, Haiping Gong, Andrew M Nesbitt

**Affiliations:** 1 UCB Pharma, Slough, UK

## Introduction

Certolizumab pegol (CZP) has a different mode of action from the other anti-TNFs, infliximab (IFX), adalimumab (ADA) and etanercept (ETA), that may be due both to its structure and how it signals through membrane TNF-α (mTNF-α). Different effects of this signalling could relate to the exact epitopes to which the anti-TNFs bind. We investigated the binding epitope for CZP and how it differs from the other anti-TNFs.

## Methods

Epitope mapping for CZP binding to TNF was achieved using minimal shift analysis of nuclear magnetic resonance) data on the non-PEGylated Fab′/TNF complex. Hydrogen/deuterium (H/D) exchange mass spectrometry was used to identify TNF peptides containing amide protons that are protected in the Fab′/TNF complex. A Biacore assay was used to determine the difference in affinity between cynomolgus monkey and human TNF for each of the 4 anti-TNFs. This technique involved the use of cross-species mutagenesis of the 4 residues that differ between cynomolgus monkey and human TNF (V37L, T44R, N72T, L138R). The epitope identified by these methods was confirmed by further site-directed mutagenesis and Biacore-based studies.

## Results

13 amino acids (E19, A22, E23, G24, Q25, Q27, L43, R44, D45, N46, Q47, I83, R138) were identified as comprising the CZP epitope on TNF. These residues formed a cluster on the outer face of each monomer. Biacore studies on TNF peptides expressed with these residues mutated showed that, although there was some overlap, the pattern of binding for each of the anti-TNFs was different.

## Conclusions

All 4 anti-TNFs tested bind to similar but not identical epitopes on the TNF molecule. These differences between the exact epitope could explain the variations in the downstream signalling properties through mTNF-α of the anti-TNFs and may help to elucidate some of the mode of action differences emerging for these agents.

